# Preconcentration of Fluorescent Dyes in Electromembrane Systems via Electrophoretic Migration

**DOI:** 10.3390/mi14020398

**Published:** 2023-02-06

**Authors:** Minsung Kim, Bumjoo Kim

**Affiliations:** 1 Department of Future Convergence Engineering, Kongju National University, Cheonan 31080, Republic of Korea; 2 Department of Mechanical and Automotive Engineering, Kongju National University, Cheonan 31080, Republic of Korea

**Keywords:** preconcentration, electrophoresis, ion-exchange membrane, fluorescent dye, microfluidics

## Abstract

Microfluidic preconcentration enables the collection or extraction of low-abundance analytes at specific locations. It has attracted considerable attention as an essential technology in bioengineering, particularly for detection and diagnosis. Herein, we investigated the key parameters in the preconcentration of fluorescent dyes based on electrophoresis in a microfluidic electromembrane system. Commercial ion-exchange membrane (IEM)-integrated polydimethylsiloxane microfluidic devices were fabricated, and Alexa Fluor 488 and Rhodamine 6G were used as fluorescent dyes for sample preconcentration. Through experimental studies, the effect of the channel concentration ratio (CCR, concentration ratio of the main and buffer channels) on the performance of the sample preconcentration was studied. The results show that the preconcentration of the target sample occurs more effectively for a high CCR or high salt concentration of the main channel when the CCR is constant. We also demonstrate a phenomenon that the salt concentration in the electrolyte solution increases as the preconcentration progresses. Our results provide consolidated conditions for electrophoresis-based sample preconcentration in electromembrane systems.

## 1. Introduction

Preconcentration is a process that involves the removal of unwanted substances, such as solvents, from chemical compounds to increase the concentration of the key solid constituents. It has been actively investigated in relation to microfluidic systems. To ensure the precise handling and detection of low-abundance analytes in a lab-on-a-chip (LOC) device with bio-samples, the development of preconcentration techniques for sample pretreatment is essential [1]. Microfluidic on-chip preconcentration is broadly classified as a static method to collect analytes at a physical obstacle or specific structure and a dynamic method based on electrokinetic equilibrium. The static method includes solvent extraction [1,2] based on phase transition and molecular diffusion; surface binding [3], which is a technique to capture analytes through bonding or adsorption; and filtration using a porous membrane or nanochannel (nanogap) [4]. The most representative techniques of the dynamic method are field amplification sample stacking (FASS) [5,6], based on the concentration gradient between the sample and the buffer; isotachophoresis (ITP) [7,8,9], to isolate analytes using an electric field based on the difference in the electrophoretic migration; and isoelectric focusing (IEF) [10,11,12], to isolate charged particles based on the difference in isoelectric points.

Recently, numerous preconcentration techniques that apply ion concentration polarization (ICP) [13], which is an intrinsic transport phenomenon observed on the surface of ion-exchange materials, have been reported. Initially, ICP-based techniques were applied by fabricating nanochannels with ion permselectivity via standard micro/nanolithography for the development of protein sample preconcentration systems based on electroosmotic flow [14]. However, to simplify the nanochannel fabrication via lithography and maximize the ICP phenomenon, microfluidic preconcentrators have been developed using ion-selective polymers (e.g., Nafion) [15]. This allows for a wide range of experimental studies for evaluating various conditions and samples to be performed with minimal device fabrication requirements and considerably enhanced preconcentration efficiency [16,17,18,19,20,21,22]. However, although the preconcentration efficiency is high for such static ICP preconcentrators, difficulties involved in extracting target samples collected at the preconcentration plug without loss significantly limit the practical application of this technology. To address this limitation, continuous ICP preconcentrators have been developed, with the addition of an independent downstream channel for the continuous separation and extraction of preconcentrated target samples. Accordingly, ICP preconcentrators based on a steady-state process have been reported to facilitate the continuous separation/preconcentration of bacterial cells or intracellular components by balancing the strong depletion force near the Nafion junction, the electrophoretic force caused by the electric field, and the drag force caused by the overall flow (external pressure driven or EOF) [23,24,25,26]. Significant efforts have been devoted to achieving a stable and high preconcentration efficiency by reducing the rapid pH shift and electrokinetic instability near the Nafion junction [27,28]. However, the Nafion-integrated ICP preconcentrator yields limited flow throughput as it comprises an inherently unscalable channel configuration. Recently reported scalable ICP preconcentrators [29,30] adopt commercial ion-exchange membranes (IEMs) instead of Nafion microfabrication to supplement the limited flow throughput. With the three-dimensional (3D) printing of a polydimethylsiloxane (PDMS) mold, a scalable fluidic channel configuration can be achieved, which allows a higher flow than that in the conventional SU8-based microfluidic system. The preconcentration of fluorescent dyes based on ICP desalination [31,32] configuration involving two IEMs with a thin, porous membrane, which separates the sample and buffer channels, has been reported [29]. The sample and buffer channels were designed asymmetrically, and different flow rates were applied to enhance the efficiency of preconcentration. Subsequently, we reported the electrophoresis-based preconcentration of submicron-scale oil droplets by fabricating a 3D printing-based PDMS microfluidic system [30]. Notably, our device is not an ICP preconcentrator as it uses both cation-exchange membrane (CEM) and anion-exchange membrane (AEM); it is based on the electrophoresis-based separation using IEMs. In our previous studies, we added two buffer channels at the top and the bottom of the main channel to prevent the introduction of chemical byproducts produced at the electrodes to the main channel (with samples). Consequently, the preconcentration efficiency was dependent on the salt concentrations at the main and buffer channels. An in-depth analysis of the difference between the absolute and relative salt concentrations in each channel and the resulting voltage and preconcentration efficiency in the main channel is yet to be conducted.

Herein, we present an effective methodology for sample preconcentration by examining various salt concentrations in the main (w/sample) and buffer (w/o sample) channels in the electromembrane system. To visualize the electrophoresis, we fabricated a scalable PDMS microfluidic device with two CEMs/AEMs and introduced two fluorescent dyes (negatively charged Alexa-Fluor 488 and positively charged Rhodamine 6G) with different polarities. It should be noted that our study aims at preconcentration of each positively and negatively charged analyte rather than separating fluorescence-labeled analytes and fluorescent dyes themselves. For this, we need to trace movement of target sample via fluorescent visualization and examine various operating parameters (e.g., voltage/current, salt concentration, etc.) as well. For an efficient comparison of the sample preconcentration with quantifiable values, we define the concentration factor (CF) based on visualization and image processing. To verify the relationship between the CF and the electrolyte concentrations and the concentration ratio between the main and buffer channels, a wide range of salt concentrations (e.g., 0.01, 0.1, 1, and 10 mM NaCl aqueous electrolyte solution) were applied to the main and buffer channels and analyzed.

## 2. Materials and Methods

### 2.1. Theory

**Figure 1:**
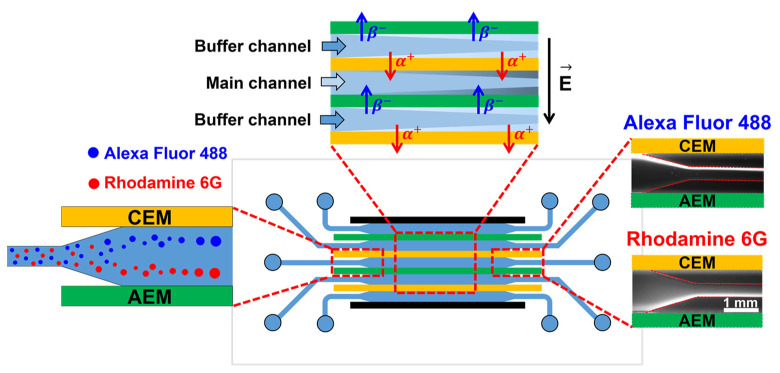
Schematic of the fluorescent dye preconcentration based on electrophoretic migration in a microfluidic electromembrane system. Voltage is applied between the top and bottom electrodes, and shear flow is applied from left to right. Migrations of Na^+^ and Cl^−^ are indicated by red and blue arrows, respectively, and the color difference in the channel depicts the ion concentration profile. Fluorescent dye added in the main channel migrates vertically depending on the electric polarity via electrophoresis and then migrates horizontally owing to the shear flow. Fluorescent images of preconcentration are shown on the right.

An IEM is a membrane that can selectively transfer specific ions. IEMs are classified into two types: AEM and CEM. When an electric field is applied across the IEM, the AEM selectively allows only anions to pass, whereas the CEM allows the passage of only cations depending on the electric polarity. In this study, as shown in Figure 1, two AEMs and CEMs were alternately placed, creating three fluidic channels whose upper and lower channel walls are composed of IEMs. In the center channel, the target sample was applied with the aqueous electrolyte solution for preconcentration; this channel was defined as the main channel. The top and bottom channels were defined as the buffer channels. When an electric field is applied to the system, charged particles in the solution migrate in specific directions depending on the electric polarity of the particle and the electric field direction, and this relative particle migration is known as electrophoresis. The particle velocity owing to electrophoresis is represented by the Smoluchowski equation [33]:

(1) $$v_{ep}=\mu_{ep}E=\frac{\varepsilon_{r}\varepsilon_{o}\zeta_{p}}{\eta }E$$

where $\mu_{ep}$ is the electrophoretic mobility; E is the applied electric field; $\varepsilon_{r}$ and $\varepsilon_{o}$ are the relative permittivity and vacuum permittivity, respectively; $\zeta_{p}$ is the zeta potential of the particle; and η is the viscosity of the electrolyte solution. When the particle is surrounded by a same solution (i.e., the same permittivity and viscosity), the main variables corresponding to particle migration are the electrophoretic mobility (i.e., zeta potential) and the electric field applied to particles. These two variables are closely related to the salt concentration of both the main and buffer electrolyte solutions. Therefore, the degree of particle preconcentration is demonstrated by changing the salt concentration of the electrolyte and the electric field. For simple interpretation, the drag acting in direct proportion to the particle velocity in the solution and the interactive repulsion between the preconcentrated particles were not considered in this study. Meanwhile, as an indicator of the effectiveness of the fluorescent preconcentration in the image analysis, the CF was defined as follows:

(2) $$\mathrm{Concentration}\;\mathrm{Factor}=\frac{1}{1-\mathrm{Recovery}}$$

Here, Recovery is the width of the particle (i.e., fluorescent dyes)-enriched stream compared to the total width of the outlet channel. The determination of the enriched boundary during image processing was performed in the same way as that in a previous study [30]. However, in this study, the CF was newly defined for obtaining a more intuitive understanding of the preconcentration level. For example, the CF was 1 in the complete absence of a preconcentration stream, and the value exceeded 1 for a high level of preconcentration.

### 2.2. Device Fabrication and Experimental Setup

**Figure 2:**
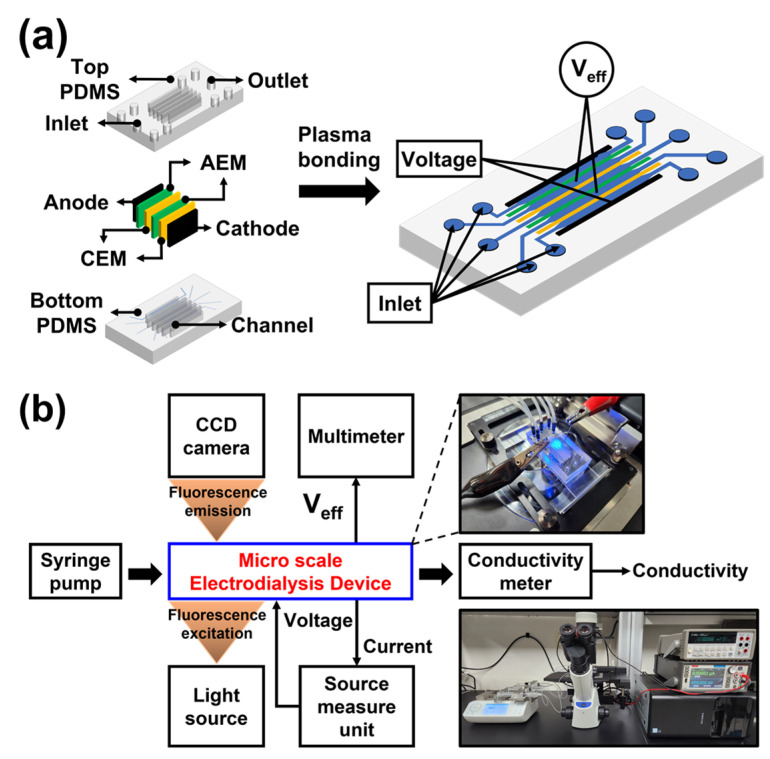
(**a**) Schematic of the fabrication of a PDMS microfluidic electromembrane device based on a 3D-printed mold for fluorescent dye preconcentration. Device was fabricated by mounting carbon electrodes, AEM, and CEM between two PDMS blocks and subsequent plasma bonding. Ag/AgCl electrodes were inserted on either side of the buffer channel in contact with the main channel to measure the unit voltage across the main channel (V_eff_). All channels of the device used in the experiment were 15 mm long, 1.5 mm wide (i.e., intermembrane distance), and 0.2 mm deep, and the width at the inlet and outlet was 0.4 mm. (**b**) Experimental setup of the microfluidic electromembrane device for fluorescent dye preconcentration. Actual photographs of the experiments are shown on the right.

Figure 2a shows the schematic of the fabrication of the PDMS microfluidic electromembrane device. As reported in previous papers, PDMS molds were fabricated via 3D printing (SLA ProJet 7000HD, 3D system, Rock Hill, PA, USA) instead of conventional SU8-based photolithography [30,32,34,35]. This is because mold structures with a high aspect ratio were necessary to accommodate commercial IEMs and electrodes in a vertical direction on the thin micro-channels. After PDMS curing, two AEM/CEM (AMHPP/CMHPP; MEGA Inc. Hodonin, Czech Republic, USD 173/m^2^) and electrodes (Spectracarb 2050A-1535; Fuel Cell Store, College Station, TX, USA) were installed between the top and bottom PDMS blocks, followed by plasma treatment for facilitating PDMS block bonding. A voltage was applied to both carbon electrodes to apply an electric field to the entire fluidic channel, and an Ag/AgCl tip was added to measure the effective voltage (V_eff_) across the main channel. All channels of the device used in the experiment were 15 mm long, 1.5 mm wide (i.e., intermembrane distance), and 0.2 mm deep, and the width at the inlet and outlet was 0.4 mm.

Figure 2b shows the experimental setup for the preconcentration of fluorescent dyes in the microfluidic electromembrane system. A source measure unit (SMU, Keithley 2460; Keithley Instruments Inc., Cleveland, OH, USA) was used to simultaneously apply the voltage and measure the current, and a shear flow (1 mm/s, 16 μL/min) was applied to the system using a syringe pump (Fusion 200-X; Chemyx Inc., Stafford, TX, USA). Additionally, a multimeter (34401A; Agilent Technologies, Inc., Santa Clara, CA, USA) was used to measure the effective voltage across the main channel, which is a key variable in electrophoresis. To estimate the preconcentration level via image analysis, a fluorescent microscope (CKX 53; Olympus, Tokyo, Japan) was used to visualize the main channel flow. To measure the salt concentration of the output flow after the preconcentration process in the main channel, a conductivity meter (Star A215 pH/conductivity meter; Orion/Thermo Fisher Scientific) and a flow-thru electrode (16–900 flow-thru conductivity electrode, Microelectrodes Inc., Bedford, NH, USA) were used to measure the conductivity at the downstream channel. The fluorescent samples added to the main channel were Alexa Fluor 488 (Alexa 488 triethylammonium; Thermo Fisher Scientific, Waltham, MA, USA) 31 μM and Rhodamine 6G (Bioreagent; Sigma-Aldrich, St. Louis, MI, USA) 28 μM in the NaCl electrolyte solution (0.01/0.1 mM). The NaCl electrolyte solutions (0.01/0.1/1/10 mM) without the fluorescent dye were used in the buffer channels.

## 3. Results and Discussion

### 3.1. Visualization of Fluorescent Dye Preconcentration

**Figure 3:**
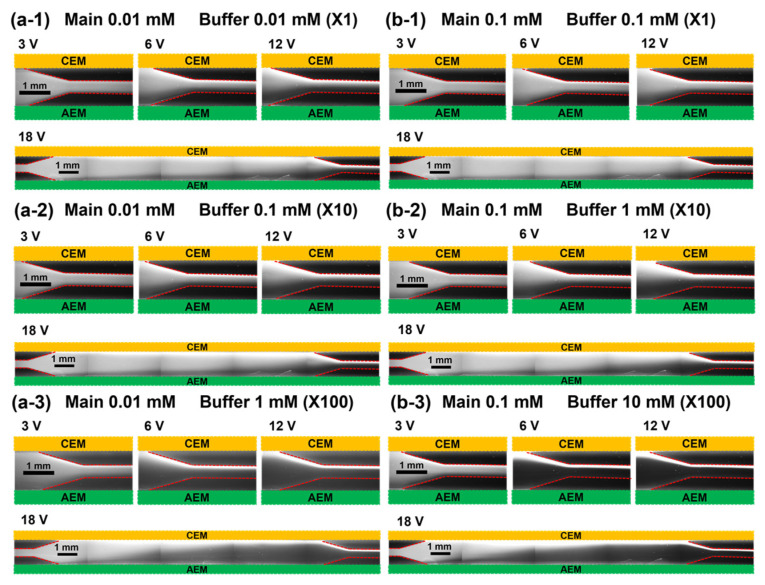
Fluorescent images of Alexa Fluor 488 preconcentration for different main channel salt concentrations of (**a**) 0.01 and (**b**) 0.1 mM sodium chloride. Bright region indicates the presence of Alexa Fluor 488. Therefore, preconcentration is better at higher voltages and buffer concentrations. Buffer concentrations are (**a-1**) 0.01 mM, (**a-2**) 0.1 mM, (**a-3**) 1 mM at main 0.01 mM and (**b-1**) 0.1 mM, (**b-2**) 1 mM, (**b-3**) 10 mM at main 0.1 mM.

**Figure 4:**
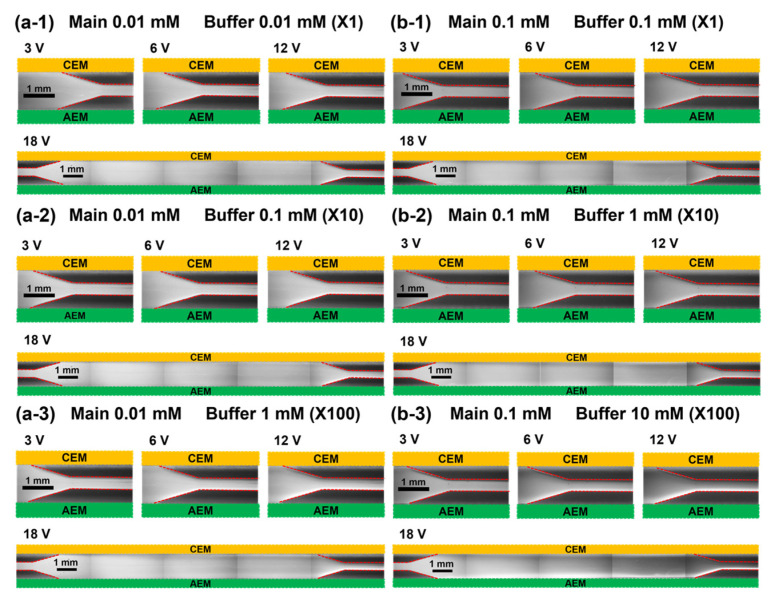
Fluorescent images of Rhodamine 6G preconcentration for different main channel salt concentrations of (**a**) 0.01 and (**b**) 0.1 mM sodium chloride. Contrary to the case of Alexa Fluor 488, Rhodamine 6G moves toward the AEM of the cathodic side owing to its electric polarity. Buffer concentrations are (**a-1**) 0.01 mM, (**a-2**) 0.1 mM, (**a-3**) 1 mM at main 0.01 mM and (**b-1**) 0.1 mM, (**b-2**) 1 mM, (**b-3**) 10 mM at main 0.1 mM.

To observe the preconcentration of fluorescent dyes for different concentration ratios between the main and buffer channels, the channel concentration ratio (CCR, *C_B_*/*C_M_* where *C_B_* and *C_M_* are the salt concentration of the buffer and the main channel, respectively) is newly defined. Here, the preconcentration experiment was conducted for cases CCR = X1, X10, and X100. Figure 3 shows the fluorescent images of the preconcentration of Alexa Fluor 488; Figure 3a,b shows the images obtained using 0.01 and 0.1 mM NaCl in the main channel, respectively. Alexa Fluor 488 moves toward the CEM on the anodic side via electrophoretic migration as it is negatively charged. Additionally, the degree of particle preconcentration is significantly higher for a higher applied voltage and CCR. This is mainly because of the increased strength of the electric field applied to the fluorescent particles. However, the determination of the degree of preconcentration for different salt concentrations of the main channel at a constant CCR value is not a straightforward method. For example, the degree of preconcentration is higher in Figure 3(a-1) than that in Figure 3(b-1), whereas the level is higher in Figure 3(b-3) than that in Figure 3(a-3). Next, the same experiments were repeated with positively charged Rhodamine 6G. Here, the electrophoretic migration is toward the AEM on the cathodic side, as shown in Figure 4. The preconcentration is better at a higher applied voltage and CCR—similar to the trend observed for Alexa Fluor 488. However, the preconcentration performance observed for Rhodamine 6G was slightly inferior to that for Alexa Fluor 488 under the same conditions. Additionally, at a constant CCR value, preconcentration is better for a higher concentration of the main channel (0.1 mM), whereas it is not clearly observed for the case of Alexa Fluor 488.

### 3.2. Calculation of CF

**Figure 5:**
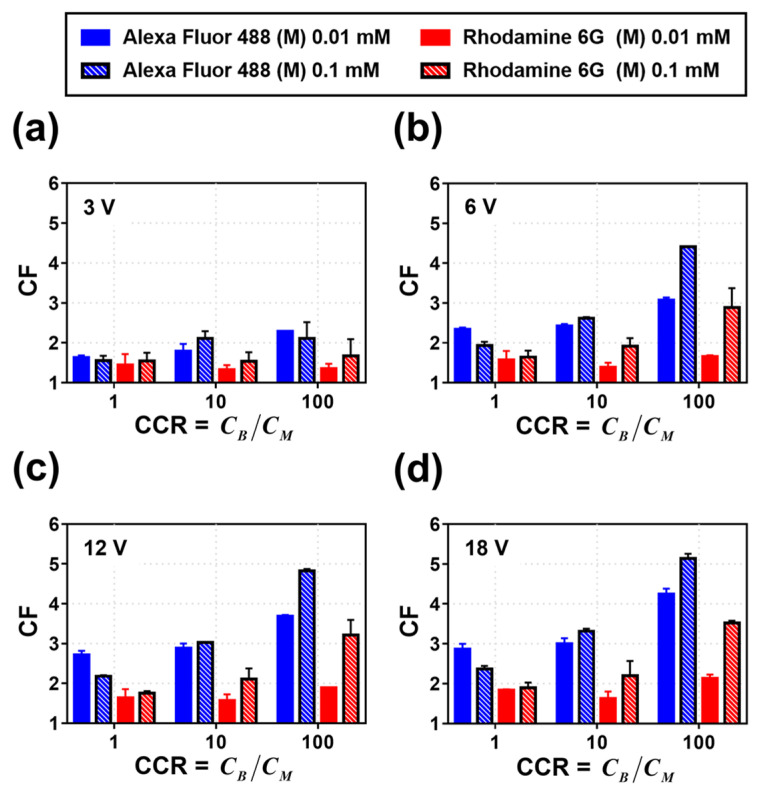
Calculation of the CF of Alexa Fluor 488 and Rhodamine 6G for CCR at (**a**) 3, (**b**) 6, (**c**) 12, and (**d**) 18 V.

Based on the visualization of fluorescent dye preconcentration, the CF was quantified through image analysis for detailed comparison. Figure 5 shows the CF for Alexa Fluor 488 (blue) and Rhodamine 6G (red) at different CCR (1, 10, 100) and applied voltage (3, 6, 12, 18 V) values. From Figure 5, the following observations can be recorded. First, the CFs (degree of preconcentration) are generally higher for Alexa Fluor 488 than those for Rhodamine 6G (${\mathrm{CF}}_{\mathrm{Alexa}\;\mathrm{Fluor}\;488}$ > ${\mathrm{CF}}_{\mathrm{Rhodamine}\;6G}$). This is attributed to the higher electrophoretic mobility of Alexa Fluor 488 than that of Rhodamine 6G, which is in agreement with previous studies [36]. Additionally, it should be considered that under neutral conditions, Rhodamine 6G is monovalent, while Alexa Fluor 488 is divalent. Second, the CF increases as the applied voltage or CCR increases. This is because the electrophoretic velocity increases as the voltage applied to charged particles increases based on Equation (1), and the proportion of the effective voltage applied to the main channel relative to the total voltage increases as the CCR increases. Third, at a constant CCR, the CF is higher for higher salt concentrations in the main channel because the conductivity of the buffer channels compared to that of the main channel offsets the increased salt concentration of the main channel; the more conductive the buffer channels, the higher the V_eff_ applied to particles. However, in the case of Alexa Fluor 488, for the same CCR (X1), the CF is higher for lower concentrations in the main channel (${\mathrm{CF}}_{M\;0.01\;\mathrm{mM}}$ > ${\mathrm{CF}}_{M\;0.1\;\mathrm{mM}}$). This is because the electrophoretic mobility of Alexa Fluor 488 increases significantly as the electrolyte concentration approaches the lowest level [37].

**Figure 6:**
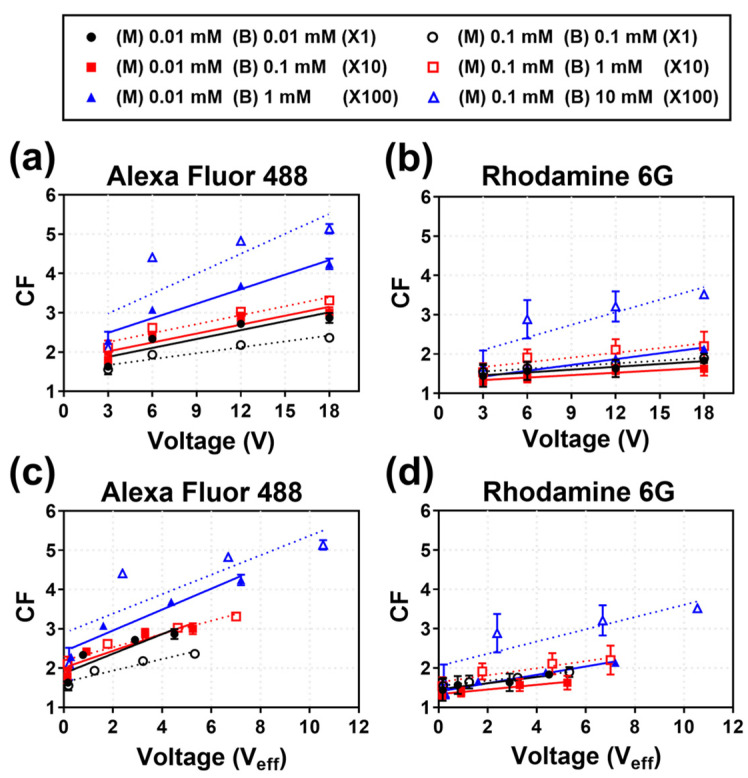
Calculation of the CF of Alexa Fluor 488 and Rhodamine 6G for different applied voltage (V) and measured effective voltage (V_eff_) values. CF depending on the applied voltage for (**a**) Alexa Fluor 488 and (**b**) Rhodamine 6G, while CF depending on the effective voltage (V_eff_) for (**c**) Alexa Fluor 488 and (**d**) Rhodamine 6G.

Following the CCR analysis, the CFs were re-arranged based on the overall voltage (V) applied between the anode and cathode and the V_eff_ measured across the main channel. Figure 6a,b show the CF for Alexa Fluor 488 and Rhodamine 6G, respectively, with respect to the applied voltage. While the total applied voltage and the CF are generally proportional, at the same applied voltage, the difference in the CFs is large, based on the salt concentration of the main/buffer channels. However, as shown in Figure 6c,d, the correlation between the V_eff_ across the main channel and the CF is linear. This is because the change in the salt concentration in the main and buffer channels is reflected in V_eff_. However, owing to technical limitations, the measured V_eff_ includes the voltage values of the main channel stream as well as the surrounding area of the main channel, although it is necessary to measure the effective voltage applied only to the main channel. In addition, the CF trend for the CCR of the Rhodamine 6G is opposite to that of Alexa Fluor 488, which is because the CF value itself is generally low due to low electrophoretic mobility of Rhodamine 6G, so that CCR (X1) and CCR (X10) come out similarly at a low level within the margin of error.

**Figure 7:**
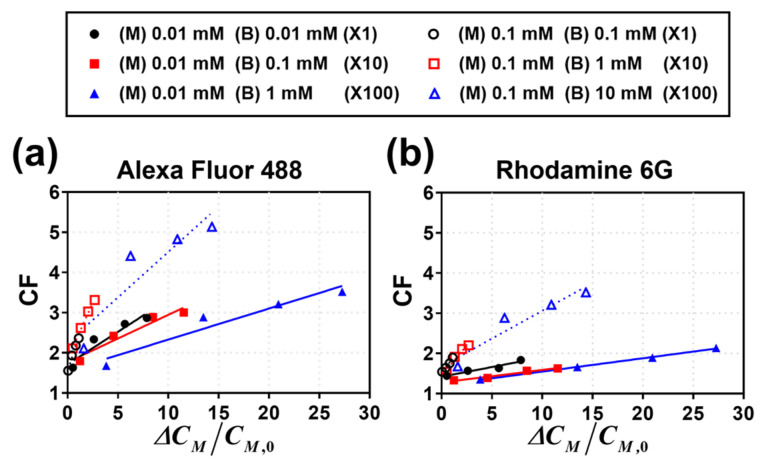
Calculation of the CF of (**a**) Alexa Fluor 488 and (**b**) Rhodamine 6G for the normalized concentration change in the main channel. Here, Δ*C_M_*/*C_M_*_,0_ is the ratio of the changed concentration value to the initial concentration.

Finally, we examined the CFs with changes in the salt concentration of the electrolyte solution. In general, as the preconcentration of the fluorescent dye in the main channel occurs, the salt concentration in the main channel increases because of the continuous ion transport through the IEM. Accordingly, the salt concentration in the electrolyte solution inevitably increases upon the preconcentration of the target sample. This was measured using a conductivity meter to identify the correlations. Figure 7a,b shows the change in CF with respect to the change in the salt concentration in the main channel for Alexa Fluor 488 and Rhodamine 6G, respectively. To enhance the CF for the target sample, a high voltage is required, which leads to a high current flow. This leads to an increase in the concentration of the electrolyte solution, thereby indicating a linear correlation between the CF and the increased salt concentration in the main channel. However, the normalized increase in the salt concentration is larger for a given CF when the salt concentration of the main channel is lower for both fluorescent dyes. Therefore, the salt concentration of the main channel should be designed to not be lowest to ensure that the target sample is preconcentrated with the maximal inhibition of the change in the electrolyte salt concentration.

## 4. Conclusions

**In this study:** experiments were conducted on the electrophoresis-based preconcentration of two well-known fluorescent dyes—the negatively charged Alexa Fluor 488 and the positively charged Rhodamine 6G. A commercial IEM-integrated microfluidic electromembrane device was fabricated and visualized. The preconcentration of the fluorescent dye was investigated by varying the salt concentration in the main channel containing the sample and the buffer channel without the sample. The following conclusions can be drawn from the findings of this study. First, the fluorescent dye preconcentration is more effective when the CCR between the main and buffer channels is higher. This is because the value of V_eff_ across the main channel with the sample increases as the CCR increases. Second, when the CCR remains constant, the fluorescent dye preconcentration is more effective for a higher main channel concentration because of the higher V_eff_ across the main channel with the higher absolute salt concentration in the buffer channel. For Alexa Fluor 488, a more effective preconcentration is possible for lower CCR and lower salt concentration in the main channel, presumably because the electrophoretic mobility is higher at lower ionic concentrations. Finally, the salt concentration in the electrolyte solution increases in the main channel as the fluorescent dye preconcentration increases, and the effect is likely to be higher for a lower solution concentration in the main channel. Based on the results of this study, we expect to achieve significant findings for a variety of bulk solutions and concentrations. Furthermore, the experiments conducted in this study can be a basis for the electrophoresis-based preconcentration of fluorescent dyes as well as other particles in electromembrane systems with IEMs.
